# Hydrogen peroxide-based products alter inflammatory and tissue damage-related proteins in the gingival crevicular fluid of healthy volunteers: a randomized trial

**DOI:** 10.1038/s41598-019-40006-w

**Published:** 2019-03-05

**Authors:** Valderlane L. P. Colares, Suellen N. L. Lima, Nágila C. F. Sousa, Mizael C. Araújo, Domingos M. S. Pereira, Saulo J. F. Mendes, Simone A. Teixeira, Cristina de A. Monteiro, Matheus C. Bandeca, Walter L. Siqueira, Eduardo B. Moffa, Marcelo N. Muscará, Elizabeth S. Fernandes

**Affiliations:** 1 Programa de Pós-graduação, Universidade Ceuma, São Luís, MA Brazil; 2 Departamento de Farmacologia, Universidade de São Paulo, São Paulo, SP Brazil; 3 School of Dentistry and Department of Biochemistry, Schulich School of Medicine & Dentistry, The University of Western Ontario, London, ON Canada; 4 Centro Universitário das Faculdades Associadas – UNIFAE, São João da Boa Vista, SP Brazil

## Abstract

Hydrogen peroxide (H_2_O_2_)-based products are effective in tooth whitening; however, their safety is controversial as they may harm patient tissues/cells. These effects are suggested to be concentration-dependent; nonetheless, to date, there are no reports on H_2_O_2_-mediated oxidative damage in the gingival tissue, and neither whether this can be detected in gingival crevicular fluid (GCF) samples. We hypothesize that H_2_O_2_ whitening products may cause collateral oxidative tissue damage following in office application. Therefore, H_2_O_2_ and nitric oxide (NO) levels were investigated in GCF samples obtained from patients undergoing dental bleaching with H_2_O_2_ at different concentrations, in a randomized, double-blind, split-mouth clinical trial. A proteomic analysis of these samples was also performed. H_2_O_2_-based whitening products promoted inflammation which was detected in GCF samples and lasted for longer following 35% H_2_O_2_ bleaching. This included time-dependent changes in NO levels and in the abundance of proteins associated with NO synthesis, oxidative stress, neutrophil regulation, nucleic acid damage, cell survival and/or tissue regeneration. Overall, H_2_O_2_-based products used in office promote inflammation irrespective of their concentration. As the inflammation caused by 35% H_2_O_2_ is longer_,_ patients may benefit better from using lower concentrations of this bleaching product, as they may result in less tissue damage.

## Introduction

Tooth whitening is currently a widely performed procedure, especially in the dental office. This is due to the increased awareness on that, aesthetically, white and well aligned teeth are standards of an ideal smile^1^. The techniques used for tooth whitening employ different whitening products which can be applied to the dental surface in varying concentrations and time of exposure^2–5^. The most commonly used whitening products are those containing hydrogen peroxide (H_2_O_2_) or carbamide peroxide at different concentrations (10–38%)^6^.

Despite the clinical efficacy of the in office whitening procedures^5^, adverse effects have been described for their usage including dental sensitivity^6–8^, soft tissue alterations^9^, genotoxicity^10,11^, pulp damage^12,13^, citotoxicity^14–16^, in addition to gingival irritation^6,17^. The inflammatory effects of tooth whitening products have also been studied; however, the evidences gathered to date are controversial^9,18–21^.

H_2_O_2_-based tooth whitening products are most frequently used in office^6,22,23^. Their efficacy is suggested to be concentration-dependent. Indeed, the higher the concentration of H_2_O_2_, the greater the occurrence of oxidative reactions, which in turn, result in the removal of tooth pigmentation^13,24–26^. Although there are no *in vivo* evidences on whether tooth whitening causes oxidative damage in the gingival tissue, *in vitro* studies have suggested that H_2_O_2_ from bleaching gels may diffuse through the enamel/dentin and thus, reduce fibroblast^27^ and odontoblast^28^ viability.

Oxidative stress occurs during inflammation as a result of an excessive generation of oxidants and/or reduced antioxidant defense mechanisms^29–31^, leading to increased tissue damage. *In vivo* produced oxidant species may be solely derived from molecular oxygen, such as H_2_O_2_, superoxide anion (${{\rm{O}}}_{2}^{-}$) and hydroxil radical (HO), being this later the most reactive and deleterious reactive oxygen species (ROS)^30^, or even result from the combined reaction with the free radical nitric oxide (NO)^30^. In fact, NO can react with ${{\rm{O}}}_{2}^{-}$ to produce peroxynitrite (ONOO^−^), which in turn may cause tissue damage by altering (oxidizing) DNA, lipids and proteins leading to the consequent loss of function^30^. The highly oxidant and bactericidal agent hypochlorous acid (HClO) is produced from myeloperoxidase (MPO), an enzyme present in the neutrophil azurophillic granules, by catalizing the oxidation of chloride (Cl^−^) anion by H_2_O_2_^32^.

We hypothesize that H_2_O_2_ whitening products may cause collateral oxidative tissue damage following in office application. Therefore, the levels of H_2_O_2_ and NO were investigated in gingival crevicular fluid (GCF) samples obtained from patients undergoing in office dental bleaching. A proteomic analysis of these samples was also performed.

## Results

### The efficacy of H_2_O_2_-containing products does not depend on the used concentration

Twenty-two of the initially recruited individuals continued through the research protocol (15% drop-off; 3 out of 25 subjects). Of note, baseline tooth shades were similar amongst individuals (7.30 ± 2.6 shade guide units (SGU)). The Table 1 shows that the commercially available H_2_O_2_-containing dental bleaching products (containing 15 and 35% H_2_O_2_) presented similar efficacy, as evaluated 7 and 21 days after the first application by the Vita Bleachedguide technique. Despite effective, the dental bleaching products did not produce differences over time, when evaluated by the Vita Classic and Vita Easyshade techniques.

**Table 1:** Clinical efficacy of tooth whitening products containing 15% or 35% H_2_O_2_.

	15% H_2_O_2_ (Mean ± SE)		35% H_2_O_2_ (Mean ± SE)	
	7 days	21 days	7 days	21 days
ΔE (Vita Easyshade)	5.9 ± 0.7	6.6 ± 0.77	5.87 ± 0.97	6.88 ± 0.6
ΔSGU (Vita Bleached)	2.22 ± 0.23	3.59 ± 0.39*	2.4 ± 0.25	4.00 ± 0.30*
ΔSGU (Vita Classic)	4.72 ± 0.46	5.27 ± 0.52	5.04 ± 0.53	5.86 ± 0.51

Clinical efficacy was determined by qualitative (Vita Classic and Vita Bleachedguide) and quantitative (spectrophotometry by Vita Easyshade). Data were collected before (baseline) and at different time-points after the bleaching procedures.

Shade guide units (SGU). Mean ± standard error (SE); *p < 0.05; differs from day 7.

### 35% H_2_O_2_ promotes greater tooth sensitivity than 15% H_2_O_2_ bleaching

Table 2 shows the effects of in office dental bleaching with H_2_O_2_ on tooth sensitivity. Both tested concentrations of H_2_O_2_ promoted tooth sensitivity; a response that was more pronounced (by 3.7-fold) following application of 35% H_2_O_2_ in comparison with 15% H_2_O_2_.

**Table 2:** Tooth sensitivity following application of whitening products containing 15% or 35% H_2_O_2_.

	15% H_2_O_2_ (Mean ± SE)		35% H_2_O_2_ (Mean ± SE)	
	1^st^ session	2^nd^ sesssion	1^st^ session	2^nd^ sesssion
1 h post-session	1.22 ± 0.45	1.10 ± 0.49	4.33 ± 0.72*	4.47 ± 0.72*
24 h post-session	0.25 ± 0.18	0.45 ± 0.31	1.54 ± 0.44	1.67 ± 0.47

Tooth sensitivity was assessed by the visual analogue scale (VAS). Data were collected at 1 h and 24 h post each bleaching procedure.

Mean ± standard error (SE); *p < 0.05; differs from the 15% H_2_O_2_ group.

### GCF samples present with higher neutrophil contents and lower ${\bf{N}}{{\bf{O}}}_{{\bf{x}}}^{-}$ concentrations after H_2_O_2_ bleaching

**Figure 1:**
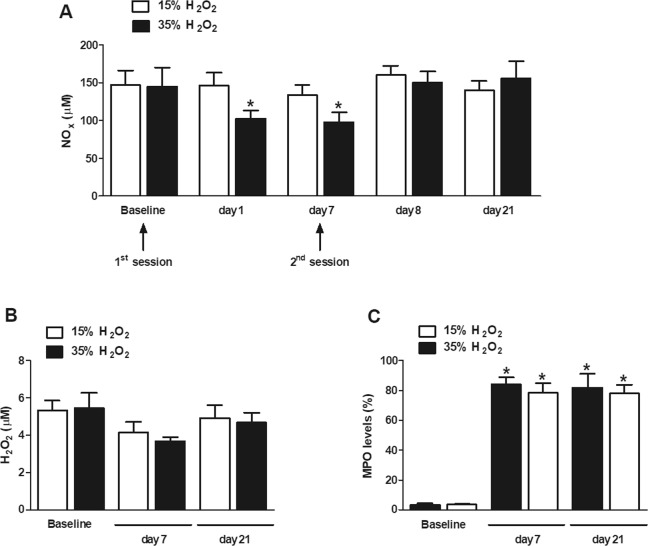
Analysis of gingival crevicular fluid markers in patients undergoing a 2-session tooth whitening with products containing hydrogen peroxide (H_2_O_2_, 15–35%). Nitric oxide end-product (${{\rm{NO}}}_{{\rm{x}}}^{-}$ = ${{\rm{NO}}}_{2}^{-}$ + ${{\rm{NO}}}_{3}^{-}$) concentrations (panel A), H_2_O_2_ concentrations (panel B) and myeloperoxidase (MPO) contents (panel C) were measured in gingival crevicular fluid samples collected before (baseline) and at different time-points after dental bleaching. *p < 0.05 vs. the corresponding baseline values.

Figure 1A depicts ${{\rm{NO}}}_{{\rm{x}}}^{-}$ concentrations measured in the GCF samples obtained before and after dental bleaching with H_2_O_2_ at 15 and 35%. Whilst no differences were observed over time after the application of 15% H_2_O_2_, the application of 35% H_2_O_2_ resulted in significant reduction (~31%) of GCF ${{\rm{NO}}}_{{\rm{x}}}^{-}$ concentrations from the day 1 to 7 after the first session. After the second bleaching session, the GCF ${{\rm{NO}}}_{{\rm{x}}}^{-}$ concentrations were similar to those detected at baseline. No significant changes were observed in GCF H_2_O_2_ concentrations due to the bleaching procedures, despite the trend to lower values observed 7 days after the first session (Fig. 1B).

Also, MPO contents were raised (22-fold increase) in GCF samples of patients who had undergone bleaching with H_2_O_2_ in comparison baseline measurements, independently of the used concentration (Fig. 1C).

### Proteomic analysis

Proteins related to NO synthesis, oxidative stress, neutrophil regulation, nucleic acid damage, cell survival and/or tissue regeneration accounted for ~25% of all detected proteins in both groups of patients over the observation period.

Proteomic analysis detected 257 proteins in baseline samples obtained from subjects exposed to 15% H_2_O_2_. Post-bleaching, there was a decline in protein abundance with subjects presenting 222 (13.6% reduction) and 154 (40.1% reduction) proteins. Amongst the detected proteins (Supplementary Table 1), there was an increase in the percentage of proteins related with NO synthesis (from 0% at baseline conditions to 1.3% at 21 days post-1^st^ session), oxidative stress (from 3.9% at baseline conditions to 5.8% at 21 days post-1^st^ session), neutrophil regulation (from 2.3% at baseline conditions to 6.5% at 21 days post-1^st^ session) and cell survival (from 9.7% at baseline conditions to 11.7% at 21 days post-1^st^ session; Table 3).

**Table 3:** Abundance of proteins associated with NO synthesis, oxidative stress, neutrophil regulation, nucleic acid damage, cell survival and tissue regeneration in GCF samples obtained from patients undergoing dental bleaching with H_2_O_2_ (15 or 35%).

		NO synthesis	Oxidative stress	Neutrophil regulation	Nucleic acid damage	Cell survival	Tissue regeneration
15% H_2_O_2_	Baseline	0%	3.9%	2.3%	3.9%	9.7%	6.6%
	7 days	1.4%	4.5%	3.2%	4.5%	10.4%	2.3%
	21 days	1.3%	5.8%	6.5%	2.6%	11.7%	6.5%
35% H_2_O_2_	Baseline	0.3%	1.7%	2.6%	2.0%	8.8%	5.4%
	7 days	0.8%	2.4%	2.8%	3.1%	8.6%	5.5%
	21 days	0.4%	2.7%	1.3%	2.2%	8.9%	3.6%

Samples were collected prior (baseline) and at different time-points after the bleaching process initiated.

Results are expressed as percentage (%) in relation with the total number of proteins detected in each time-point.

On the other hand, the same samples presented with a transient increase of proteins related with nucleic acid damage (from 3.9% at baseline conditions to 4.5% at 7 days post-1^st^ session), with this group of proteins representing 2.6% of the total proteins detected at the end of the observation period (21 days post-1^st^ session; Table 3). Similarly, there was a transient reduction in the percentage of proteins associated with tissue regeneration, as 6.6% were observed in pre-bleaching conditions and 2.3% and 6.5% were registered at days 7 and 21 post-1^st^ session, respectively (Table 3).

The GCF samples obtained from patients submitted to 35% H_2_O_2_ bleaching were also evaluated. From the 353 proteins detected at pre-bleaching conditions, 255 (27.8% reduction) and 225 (36.3% reduction), were observed for days 7 and 21 post-1^st^ session. Of the detected proteins (Supplementary Table 2), a progressive increase was observed in the percentage of proteins associated with oxidative stress (from 1.7% at baseline conditions to 2.7% at 21 days post-1^st^ session). The percentage of proteins associated with neutrophil regulation was stable until the 7^th^ day post-1^st^ session of bleaching, diminishing after 21 days (from 2.6% at baseline conditions to 1.3% at 21 days post-1^st^ session). A similar profile was noted for proteins involved in tissue regeneration (from 5.4% at baseline conditions to 3.6% at 21 days post-1^st^ session; Table 3).

On the contrary, the same samples exhibited a transient increase of proteins associated with damage of nucleic acids from 2.0% at baseline conditions to 3.1%, 7 days post-1^st^ session, with their levels returning to 2.2% at the end of the observation period (21 days post-1^st^ session) (Table 3). A similar response was observed for proteins associated with NO production, with these proteins representing 0.3% of the detected proteins at baseline conditions, and then, 0.8% and 0.4%, at days 7 and 21 post-1^st^ session, respectively (Table 3). Proteins associated with cell survival remained stable throughout the bleaching procedure (8.6–8.9%; Table 3).

**Figure 2:**
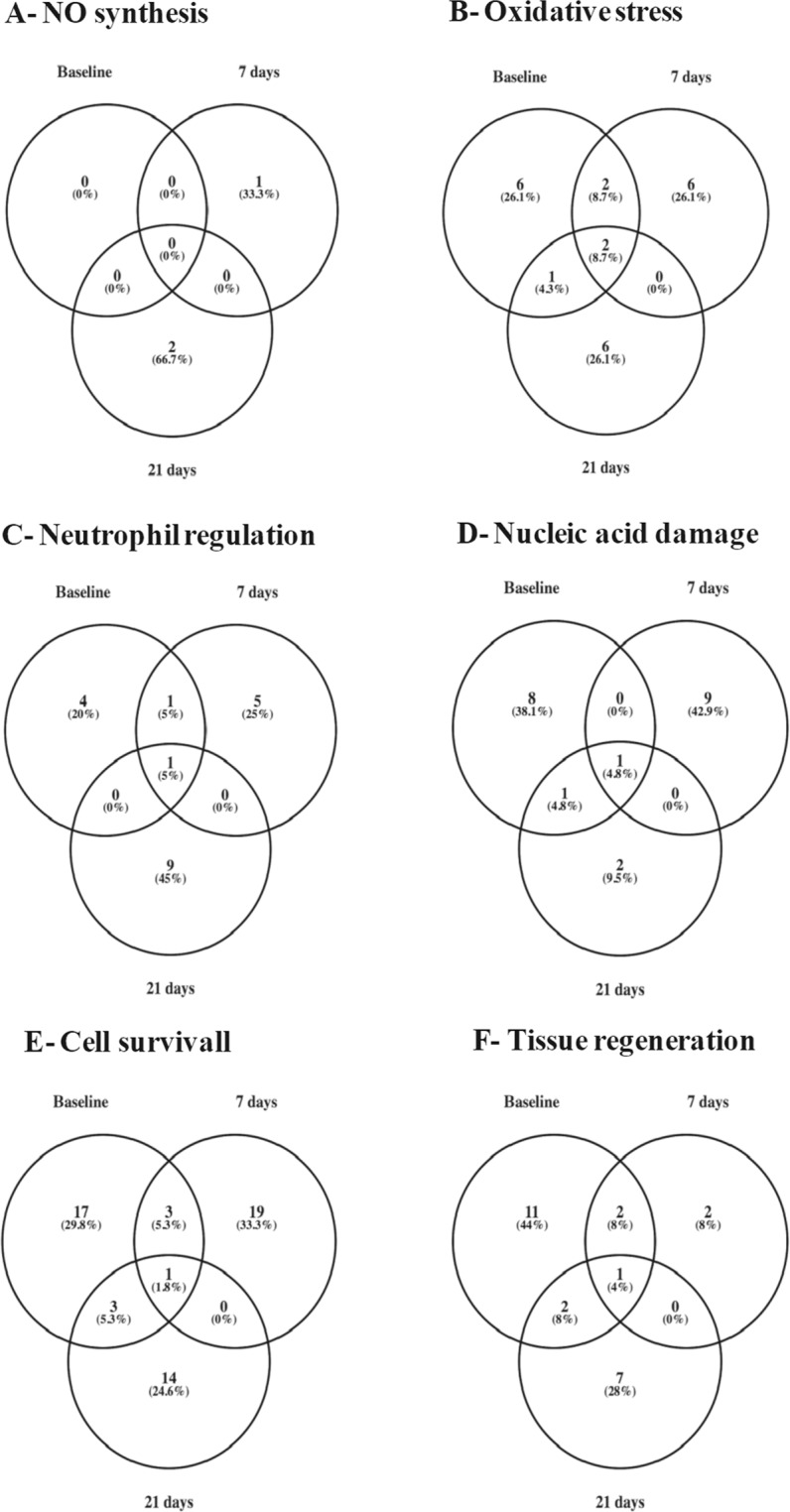
Time-dependent abundance of proteins detected in gingival crevicular fluid samples obtained from patients undergoing a 2-session tooth whitening with hydrogen peroxide (H_2_O_2_, 15%). Proteins were classified by biological function as associated with: NO synthesis, oxidative stress, neutrophil regulation, nucleic acid damage, cell survival and tissue regeneration. Samples were collected before (baseline) and at different time-points after dental bleaching.

**Figure 3:**
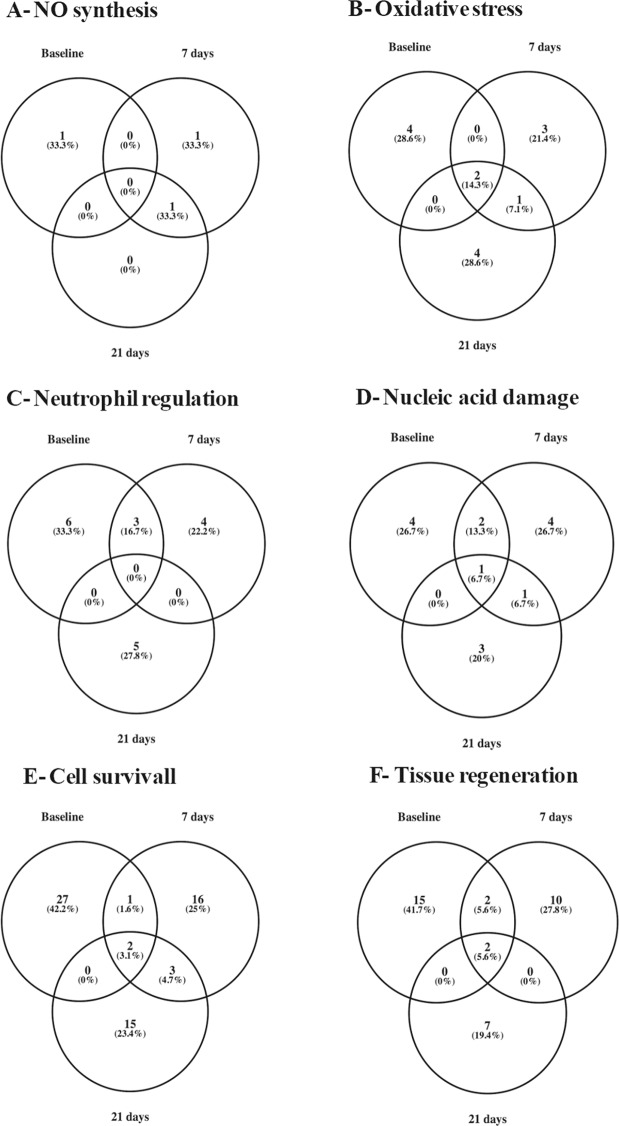
Time-dependent abundance of proteins detected in gingival crevicular fluid samples obtained from patients undergoing a 2-session tooth whitening with hydrogen peroxide (H_2_O_2_, 35%). Proteins were classified by biological function as associated with: NO synthesis, oxidative stress, neutrophil regulation, nucleic acid damage, cell survival and tissue regeneration. Samples were collected before (baseline) and at different time-points after dental bleaching.

Amongst the evaluated classes of proteins, some of them participate in different processes, as observed in Supplementary Tables 1 and 2. The Venn diagrams (Figs 2 and 3), show the dynamics of the detected proteins over the observation period, with different proteins participating in a process at specific time-points.

## Discussion

Previous studies have shown that H_2_O_2_-containing tooth whitening products present similar efficacy irrespective of the concentration used^5^. Here, the efficacy of the products tested was confirmed by Vita Bleachedguide, Vita Classic and Vita Easyshade analysis, although time-dependent effects were only observed by using the Vita Bleachedguide technique. Indeed, this analysis showed a greater efficacy 21 days after the first session.

Although effective, the safety of the application of H_2_O_2_ gels is of debate, as deleterious effects in the patient tissues/cells have been suggested for these products, including dental sensitivity^6–8^, cytotoxicity^14–16^ and genotoxicity^10,11^, pulp damage^12,13^ and gingival irritations^6,17^, amongst others. Evidences also suggest that inflammation may occur following the application of dental bleaching, specially at pulp level^9,18,19,21,33^. These effects are suggested to be concentration-dependent; however, to date, there are no reports on H_2_O_2_-mediated oxidative damage in the gingival tissue, or neither whether this may be relevant enough to be detected in a timely manner in GCF samples.

In this study, we show for the first time that tooth whitening products containing H_2_O_2_ promote pro-inflammatory alterations able to be detected in GCF samples collected by following a simple and non-invasive procedure. The GCF is an exudate rich in serum, leukocytes, structural cells and microorganisms, considered as a potential indicator of an ongoing inflammation and monitoring tool post-dental procedures^34–36^. Of note, GCF samples composing the test groups presented different amounts of proteins at baseline conditions. Although obtained from the same group of individuals, it is possible that daily dental care influences the composition of GCF; for instance, one may exert different forces when brushing different sides of the mouth. Indeed, mechanical stimuli such as vigorous brushing are known to affect the volume and composition of GCF samples^37^.

The ability of H_2_O_2_-based bleaching gels to cause inflammation is not novel. Indeed, previous studies demonstrated that the in office application of high concentrations of H_2_O_2_ (35–38%) causes pulp inflammation characterized by leukocyte migration, angiogenesis and tissue damage^21,33^, and that this effect is concentration-dependent with less damage occurring with the lowest concentrations of the bleaching product. On the other hand, evidences on gingival inflammation following in office tooth whitening are controversial, as this effect may depend on exposure conditions to H_2_O_2_ such as trays and use of light during the procedure^19,38^. Also, a recent report using a similar bleaching protocol to the one used herein, analyzed the levels of cytokines in GCF samples; the study suggested that H_2_O_2_-based gels do not cause inflammation at GFC level^20^. In this study, a significant reduction in the GCF ${{\rm{NO}}}_{{\rm{x}}}^{-}$ concentrations was detected only in patients who underwent bleaching with 35% H_2_O_2_. This effect was observed as soon as 1 day after the first dental bleaching session and remained low during the next 7 days. On the other hand, after the 2^nd^ session, these concentrations returned to those observed at baseline conditions. A similar profile, although less pronounced and of no statistical significance, was observed for GCF H_2_O_2_ concentrations for both bleaching products. On the contrary, GCF neutrophil contents (as assessed by MPO levels) were raised in patients undergoing bleaching, irrespective of either the H_2_O_2_ concentration or the time of evaluation. These data indicate that, at the 15–35% range concentration, H_2_O_2_ causes inflammation; however, the mediators and pathways involved in this response may depend on its concentration.

The proteomic analysis of the GCF samples showed that dental bleaching causes alterations in the abundance of proteins associated not only with NO and H_2_O_2_ synthesis, but also with neutrophil recruitment. Proteins associated with NO synthesis and oxidative stress were increased following bleaching, irrespective of H_2_O_2_ concentration.

Mitochondrial NADH dehydrogenase ubiquinone flavoprotein 3, involved in NO synthesis, was detected in samples obtained from of both groups of bleaching. Other proteins related to NO synthesis such as mitogen-activated protein kinase kinase kinase 4, NADH-ubiquinone oxidoreductase chain 2, mitochondrial NADH dehydrogenase ubiquinone flavoprotein 3 and TIR-domain containing adaptor molecule 1 were found in GCF samples following tooth whitening with 15% H_2_O_2_; whilst NADH-ubiquinone oxidoreductase subunit 5 was only detected in those who received 35% H_2_O_2_.

Oxidoreductases such as JHDM1D, NADH-ubiquinone oxidoreductase chain 2, NADH-ubiquinone oxidoreductase subunit 5, NADH dehydrogenase [ubiquinone] flavoprotein 3, dehydrogenase/reductase 2-isoform 2, retinol dehydrogenase 14, epydidimal secretory protein Li 55, mitochondrial peroxiredoxin-5 and EGLN3, and the antioxidant protein S100-A8 were detected in GCF samples at the day 7 post-1^st^ session with H_2_O_2_. Despite the detection of oxidoreductases such as cytocrome P450 2D6, NADH dehydrogenase ubiquinone flavoprotein 3, EGLN3, the epydidimal secretory protein Li 55 and the antioxidant protein S100-A8; at 21 days post-1^st^ session, the samples were also positive for annexin and transient receptor potential channels M2 and V1, proteins involved in the sensing and production of oxidant molecules^39–42^. This alteration in the expression of regulators of NO synthesis and oxidative stress, explains, at least in part, the profile of NO and H_2_O_2_ release in patients after bleaching.

Different proteins that regulate neutrophil chemotaxis, activation and degranulation were detected in GCF samples in both pre- and post-bleaching conditions. The largest abundance of proteins of these classes was observed for patients who had undergone 15% H_2_O_2_, 21 post-1^st^ session. GCF samples obtained from patients treated with either 15 or 35% H_2_O_2_ presented proteins associated with the activation and degranulation of neutrophils (keratin 1, S100-A8, eosinophil cationic protein, neutrophil defensin, tyrosine-phosphatase beta receptor-like, TIR-domain containing adaptor molecule 1 and resistin) at this time point; however, proteins related with neutrophil chemotaxis and death (inositol-trisphosphate 3-kinase B and annexin) were only detected in the 15% H_2_O_2_ bleaching treatment. Pre-bleaching conditions presented proteins associated with neutrophil migration, activation and degranulation (beta-defensin 119, NF-kappa-B subunit p105, kinase serine/threonine protein 10, hornerin, calcium/calmodulin-dependent kinase type 1D, Ig variable chain 3–11, S100-A8, maltase-glucoamylase, phospholipid-transporting ATPase 8AI, N-acetylgalactosamin-6-sulphatase and N-acylesphingosine amidohydrolase 1).

For both H_2_O_2_ concentrations tested, there was an increase in the abundance of proteins related with nucleic acid damage, 7 days post-1^st^ session, but an increase in the percentage of cell survival proteins was only observed following 15% bleaching in comparison with pre-whitening conditions. Tissue regeneration proteins were reduced at 7 days for the 15% and 21 days for the 35% bleaching.

Twenty one days following the 1^st^ session of bleaching, there was a restoration in the percentage of GCF proteins related to tissue regeneration (angiogenesis, re-epithelialization, fibroblast proliferation, neuronal regeneration, osteoclastogenesis and dentin production) only in those undergoing bleaching with the lowest concentration of H_2_O_2_. These evidences allow us to suggest the existence of an inflammatory process that is more exacerbated following tooth whitening with 35% H_2_O_2_, and lasts for the whole observation period.

According to these findings (NO and H_2_O_2_ levels and proteome), and considering that both NO and H_2_O_2_ are constitutively produced by endothelial cells, neurons and/or keratinocytes^43–45^, we can suggest that the 35% H_2_O_2_ bleaching may damage NO-producing cells (neurons and endothelial cells) and that both concentrations of H_2_O_2_ cause damage in H_2_O_2_-producing cells such as keratinocytes 7 days post-1^st^ session. The observed reduction in NO levels at this time-point may be also due to its ability to react with with ${{\rm{O}}}_{2}^{-}$ to produce peroxynitrite, which in turn, causes cell damage^30^.

In later time-points (21 days post-1^st^ session), it was observed the restoration of the crevicular levels of NO and H_2_O_2_; this can be related to: i) increased numbers of cell survival and tissue regeneration proteins, in addition to increased activation of neutrophils with 15% bleaching; and ii) ongoing neutrophil activation and restoration of proteins associated with NO synthesis, and in parallel, a reduction in the abundance of regenerative proteins, following bleaching with either gel products.

Of note, evaluation of dental sensitivity demonstrated that a greater sensitization occurs following 35% in comparison with 15% H_2_O_2_ bleaching; a response that was of similar magnitude following both bleaching sessions. The mechanisms of dental sensitivity are not fully understood; however, damage of dental pulp cells following H_2_O_2_ has been associated with increased tooth sensitivity^12,13,27^. Also, sensory nerve endings were previously found in areas of the inner dentin which are close to the pulp^46,47^. Thus, it is possible that products generated/released during H_2_O_2_-induced pulp damage contribute to tooth hypersensitivity following tooth whitening.

Overall, our results show that in office tooth whitening procedures employing H_2_O_2_ present similar efficacy, however, they cause inflammation irrespective of the used concentration. As the inflammation caused by 35% H_2_O_2_ lasts for longer, patients may benefit better from using lower concentrations of this bleaching product, as they may result in less tissue damage.

## Methods

### Patients

The study was reviewed and approved by the Human Research Ethics Committee of the Universidade CEUMA (protocol number 1.307.220) and was performed in accordance with the Declaration of Helsinki 1975, as revised in 2008. The study was registered under the Brazilian Register of Clinical Assays (protocol number RBR-4kkcd7, registered in 05/11/2015, as “Evaluation of fluid gingival after bleaching with hydrogen peroxide in different strengths: clinical study randomized”). The primary outcome of the study was the tooth shade evaluation. Power analysis indicated that 22 patients were required in order to achieve a 90% chance of detecting a decrease in tooth shade by at least 2 SGU in comparison with baseline measurements (α = 0.05). Sample size was calculated on the website www.sealedenvelope.com.

A total of 25 healthy subjects (18–40 years old) presenting pigmented (colour equal to or darker than A3) upper anterior teeth without sensitivity, were recruited for participation in the study. Tooth sensitivity to cold was evaluated as previously described^20^ by using the visual analogue scale (VAS; from 0–10; with 0 = no sensitivity and 10 = maximum sensitivity), and it was also registered at 1 h and 24 h post each bleaching session. All subjects presented good general and oral health and hygiene, with no history of periodontal disease or gingival retractions, dental restorations or carious lesions, bruxism, dental sensitivity and had not recently taken analgesic and/or anti-inflammatory drugs. Smokers, pregnant or lactating women, those who had previously undergone tooth whitening or endodontic treatment or had dental prosthesis or orthodontic appliances in the upper anterior teeth were excluded from the study. A written informed consent was obtained from each participant. A prophylaxis was performed in each participant on the week just prior to the beginning of the tooth whitening procedure.

### Experimental groups

This study was a randomized, double-blinded, split-mouth clinical trial. The mouth of each participant was split with an equal allocation rate of 1:1 for one of two treatments (H_2_O_2_, 15 and 35%; block of 2). For this, treatments were labeled A and B, and were randomically assigned to each side of mouth of each subject by computer-generated tables prepared by a third person not involved in the research protocol (www.sealedenvelope.com). Both the participants and the experimenters were blinded to the protocol. Details of the allocated group were recorded on cards contained in sequentially numbered, opaque, sealed envelopes. The envelopes were opened by the patients on the day of bleaching to prevent disclosure of the randomization scheme. All gels were prepared according to the manufacturer´s instructions by a third person who handled over to the operator, the gels according to their previous allocation as A or B. The study was conducted at the clinic of the School of Dentistry of Local University from March 2016 to August 2016; patients were recruited prior to the procedure, in March and April 2016.

### Tooth whitening procedure

After prophylaxis, the initial color was registered and then, the bleaching procedure was performed. Before applying the whitening gel, the gingival tissue of the upper anterior teeth was isolated using a light-polymerized resin dam (Top Dam; FGM Prod. Odontol. Ltda., Joinville, SC, Brazil). The H_2_O_2_-containing bleaching products (Lase Peroxide Lite 15% and Lase Peroxide Sensy 35%; DMC Equip., São Carlos, SP, Brazil) were applied for 15 min, three times in each session, without using light. Two bleaching sessions were performed with a 1-week interval between them. Participants were instructed to brush their teeth regularly using toothpaste without a desensitizing or bleaching agent. Bleaching efficacy was determined by comparison between the initial color shade and the one achieved post each session; by using the qualitative Vita Classic and Vita Bleachedguide 3D (Vita Zahnfabrik, Bad Säckingen, Germany) and the quantitative Vita Easyshade spectrophotometry (Vident, Brea, CA, USA) techniques. All patients were evaluated over 21 days post first session. Efficacy results were calculated as previously described^20^.

### Collection of gingival crevicular fluid samples

Gingival crevicular fluid (GCF) samples were collected for analysis. For this, the soft tissues were isolated with cotton rolls and the upper anterior teeth were gently dried with air for 5–10 s. The GCF was collected from the teeth number 11 and 21, by using Perio-paper strips (IDE Interstate, Amityville, NY, USA). Two strips were individually inserted into the sulcus of each tooth (1–2 mm depth), for 60 seconds. Strips containing blood were discarded and a novel sample was then collected from a different site of the same tooth. After sample collection, strips were immediately placed in sterile Eppendorf tubes and the GCF samples were stored at −80 °C for further analysis. Samples were collected prior to (baseline), and 7 and 21 days after the beginning of the dental bleaching procedure.

### Biochemical analysis of GCF samples

#### Sample preparation

Paper strips containing GCF samples (1 from each tooth) were incubated with 160 μl of phosphate-buffered saline (PBS) for 10 min, with vortexing at every 2 min of incubation. Then, samples were centrifuged at 1.200 × g, for 5 min. The resulting supernatants were collected and kept at −80 °C for further analysis of total ${{\rm{NO}}}_{2}^{-}$ + ${{\rm{NO}}}_{3}^{-}$ concentration (${{\rm{NO}}}_{{\rm{x}}}^{-}$; final products of NO oxidation), H_2_O_2_ and MPO levels (as a measurement of neutrophil contents).

#### Quantification of total NO_x_

Total ${{\rm{NO}}}_{{\rm{x}}}^{-}$ (${{\rm{NO}}}_{2}^{-}$/${{\rm{NO}}}_{3}^{-}$) concentrations were measured by the Griess reaction method for ${{\rm{NO}}}_{2}^{-}$ after the nitrate reductase-catalysed reduction of ${{\rm{NO}}}_{3}^{-}$ to ${{\rm{NO}}}_{2}^{-}$, as previously described^48^. For this, 80 µl of sample were incubated with 20 µl of 1 U/ml nitrate reductase (Sigma-Aldrich) and 10 µl of 1 mM NADPH (Sigma-Aldrich) for 30 min at 37 °C, in a 96-well plate. Then, 100 µl of Griess reagent (Sigma-Aldrich) were added and incubated for 15 min at 37 °C. Absorbance was measured at 550 nm immediately using a microplate reader (MB-580; Heales, Shenzhen, China). After subtraction of background readings, the absorbance in each sample was compared with that obtained from a sodium nitrite (0–100 μM) standard curve. Results are expressed as ${{\rm{NO}}}_{{\rm{x}}}^{-}$ concentrations in µM.

#### Quantification of H_2_O_2_ concentrations

H_2_O_2_ concentrations in the GCF samples were measured by using a H_2_O_2_/peroxidase assay kit (Amplex Red H_2_O_2_/Peroxidase assay kit; Molecular Probes, Invitrogen), as previously described^48^. Briefly, 50 μl of the diluted GCF samples were incubated with 50 μl of a solution containing 0.05 M NaH_2_PO_4_ (pH 7.4), 0.2 U/ml horseradish peroxidase (HRP) and 25.7 mg/ml of the Amplex Red (10-acetyl-3,7-dihydroxyphenoxazine) reagent, during 2 h at 37 °C. After the incubation, the absorbance of the reaction mixture was read at 560 nm. Absorbance readings were compared with those obtained from a H_2_O_2_ standard curve (0–40 µM). Results are expressed as H_2_O_2_ concentrations in µM.

#### Measurement of MPO activity

MPO activity in GCF samples was assessed as an index of neutrophil influx, by measuring the speed of oxidation of *o*-dianisidine in the presence of H_2_O_2_^49^. Samples (25 µl) were added of an equal volume of a potassium phosphate solution (5 mM; pH 6.0) containing 0.5% HTAB (Sigma Chem. Co, USA). Then, the samples were sonicated for 20 s and incubated at 60 °C for 2 h, for inactivation of endogenous catalase. Then, samples were centrifuged at 10,000 g for 5 min. An aliquot (10 µl) of the supernatant was incubated with 200 µl of potassium phosphate (pH 6) containing 16.7 mg/ml *o*-dianisidine (Sigma Chem. Co., EUA) and 0.0005% H_2_O_2_ per well in a 96-well plate. The speed of the formation of the oxidation product of *o*-dianisidine was read at 460 nm every 10 s for 10 min. MPO levels were calculated by comparing the initial and final registered absorbance. The results are expressed as the percentage (%) of maximum speed of reaction in comparison with the initial absorbance of each sample.

### Proteomic analysis

#### Sample preparation

For proteomic analysis, a pool of strips (1 from each tooth) obtained prior to and from each time-point after exposure with the different bleaching products, were incubated with 150 µl of a solution containing 80% acetonitrile, 19.9% distilled water and 0.1% trifluoroacetic acid, and were then, sonicated 3 times for 1 min. Samples were dried and ressuspended in 1000 µl of distilled water. Total protein concentration was then, determined in each pool of samples by using the Micro BCA assay kit (Thermofisher, USA). Aliquots containing 10 µg/pool of sample were dried, denaturated and reduced by addition of 200 µl of buffer 1 (containing 4 M urea, 10 mM DTT and 50 mM NH_4_HCO_3_; pH 7.8). After 2 h, samples were added of 1000 µl of buffer 2 (50 mM NH_4_HCO_3_, pH = 7.8) and the proteins were digested for 18 h at 37 °C, with 2% (w/w) sequencing-grade trypsin (Promega, Madison, WI, USA), samples were desalted (Zip Tip C-18, EMD Millipore Inc., Germany) and submitted to mass spectrometric analysis after HPLC separation (LC-ESI-MS/MS).

#### Liquid cromatography by mass spectrometry

Samples were resuspended in solvent A (97.5% distilled water/2.4% acetonitrile /0.1% formic acid) and then subjected to RP nLC-ESI-MS/MS, using a LTQ-Velos (Thermo Scientific, San Jose, CA, USA) mass spectrometer. LC aligned with the C18 column of capillary-fused silica (column length 10 mm, column id 75 m, 3 m spherical beads, and 100 A° pores size) was used, linked to the MS through electrospray ionization (ESI). The survey scan was set in the range of m/z values 390–2000 MS/MS. Peptides were eluted from the nanoflow reversed phase-high-performance liquid chromatography (RP-HPLC) over a 65 min period, with linear gradient ranging from 5 to 55% of solvent B (97.5% acetonitrile, 0.1% formic acid), at a flow rate of 300 nl/min, with a maximum pressure of 280 bar. The electrospray voltage was 1.8 kV and the temperature of the ion-transfer capillary was 300 °C. After a MS survey scan range within m/z 390–2000 was performed and after selection of the most intense ion (parent ion), MS/MS spectra were achieved via automated sequential selection of the seven peptides with the most intense ion for collision-induced dissociation (CID) at 35% normalized collision energy, with the dynamic exclusion of the previously selected ions. The MS/MS spectra were matched with human protein databases (Swiss-Prot and TrEMBL, Swiss Institute of Bioinformatics, Geneva, Switzerland, https://ca.expasy.org/sprot/) using SEQUEST algorithm in Proteome Discoverer 1.3 software (Thermo Scientific, USA). The searches were performed by selecting the following SEQUEST parameters: (1) trypsin as protease enzyme, (2) 2 Da precursor ion mass tolerance, (3) 0.8 Da fragment ion mass tolerance, and (4) dynamic modifications of oxidized cysteine and methionine and phosphorylated serine and threonine. A maximum of four dynamic modifications per peptide were accepted. The SEQUEST score filter criteria applied to the MS/MS spectra for peptides were absolute XCorr threshold 0.4, fragment ion cutoff percentage 0.1, and peptide without protein XCorr threshold 1.5. Any nontryptic peptides passing the filter criteria were discarded. Only proteins for which two or more peptides were identified are reported in this study.

Protein functions were assessed in the UniProt protein (https://www.uniprot.org/) and the National Center for Biotechnology Information (NCBI) gene (https://www.ncbi.nlm.nih.gov/) data banks, and literature search.

### Statistical analysis

The results are presented as the mean ± standard error (SE). Statistical comparison was performed by analysis of variance of repeated measures followed by the Bonferroni test. p < 0.05 were considered significant.

## Supplementary information

Supplementary Tables 1 and 2

## Data Availability

The datasets used to support this study will be made available upon reasonable request. Requests should be sent to the corresponding author.
