# Supplementary material for: Hydrogen peroxide-based products alter inflammatory and tissue damage-related proteins in the gingival crevicular fluid of healthy volunteers: a randomized trial
Source: Sci Rep. 2019 Mar 5;9:3457. doi: 10.1038/s41598-019-40006-w (PMC6400941; doi:10.1038/s41598-019-40006-w)
Supplement: Supplementary file 1 — Supplementary Tables 1 and 2 [file 41598_2019_40006_MOESM1_ESM.pdf]

Hydrogen peroxide-based products alter inflammatory and tissue damage-related proteins in the gingival crevicular fluid of healthy volunteers: a randomized trial

Valderlane L.P. Colares[1], Suellen N.L. Lima[1], Nágila. C.F. Sousa[1], Mizael C. Araújo[1], Domingos M.S. Pereira[1], Saulo J.F. Mendes[1], Simone A. Teixeira[2], Cristina de A. Monteiro[1], Matheus C. Bandeca[1], Walter L. Siqueira[3], Eduardo B. Moffa[1,3], Marcelo N. Muscará[2], Elizabeth S. Fernandes[1#]

Supplementary Table 1. Identification of proteins associated with NO synthesis, oxidative stress, neutrophil regulation, nucleic acid damage, cell survival and tissue regeneration in GCF samples obtained from patients undergoing dental bleaching with 15% H_2O_2. Samples were collected prior (baseline) and at different time-points after the bleaching process initiated.

Protein	Baseline						7 days						21 days					
	NO synthesis	Oxidative stress	Neutrophil regulation	Nucleic acid damage	Cell survival	Tissue regeneration	NO synthesis	Oxidative stress	Neutrophil regulation	Nucleic acid damage	Cell survival	Tissue regeneration	NO synthesis	Oxidative stress	Neutrophil regulation	Nucleic acid damage	Cell survival	Tissue regeneration
**5' exonuclease Apollo**				+													+	
**Abnormal spindle-like microcephaly-associated protein**					+												+	
**Active regulator of SIRT1**					+						+							
**Adenomatous polyposis coli protein**											+							
**Alpha-ketoglutarate-dependent dioxygenase FTO**		+		+														
**Alstrom syndrome 1, isoform CRA_c**																	+	
**Ankyrin repeat and LEM domain-containing protein 2**					+													
**Annexin**														+	+			+
**Annexin-2 receptor**											+							
**Apolipoprotein A-I**																		+
**ATP-binding cassette sub-family A member 12**						+												
**ATP-dependent DNA helicase**										+								

**Autophagy-related protein 16-1**					+						+							
**BM-016**										+	+							
**Breast and ovarian cancer susceptibility protein 2 truncated variant**										+	+							
**Cannabinoid receptor 1**		+																
**CD45**									+									
**cDNA FLJ14215 fis, clone NT2RP3003665, highly similar to Beta-ureidopropionase (EC 3.5.1.6)**													+					
**cDNA FLJ35792 fis, clone TESTI2005759, highly similar to Amphoterin-induced protein 2**					+												+	
**cDNA FLJ52316, highly similar to NADPH--cytochrome P450 reductase**								+										
**cDNA FLJ52401, highly similar to Integrin alpha-10**															+			
**cDNA FLJ54646**														+				
**cDNA FLJ55119, highly similar to Homo sapiens ZW10 interactor (ZWINT), transcript variant 3, mRNA**											+							
**cDNA, FLJ94365**											+							
**Centriolin**																	+	
**Collagen alpha-6(VI) chain**						+												
**Cyclin-G2**					+													
**Cytochrome P450 2D6**														+				
**Cytoskeleton-associated protein 5**											+							
**Dehydrogenase/reductase (SDR family) member 2 isoform 2 variant**								+										
**Disintegrin and metalloproteinase domain-containing protein 8**					+													
**DNA ligase 1**					+													

**Dual-specificity protein phosphatase 13 isoform B**																	+	
**Dynactin subunit 1**											+							
**Dynactin subunit 2**																	+	
**E3 SUMO-protein ligase RanBP2**																	+	
**E3 ubiquitin-protein ligase RBBP6**										+								
**EGLN3 protein**		+						+						+				
**Elongation factor 1-alpha 2**					+												+	
**Eosinophil cationic protein**															+			
**Fanconi anemia group D2 protein**								+		+								
**Folliculin-interacting protein 2**																+	+	
**Frizzled-9**												+						
**G2/mitotic-specific cyclin-B3**																	+	
**GRAM domain-containing protein 4**					+													
**Heat shock protein beta-7**					+													
**Histone acetyltransferase p300**				+	+													
**Histone cluster 1, H1t**				+						+						+		
**Histone H2A-Bbd type 1**																+		
**Histone H4**				+														
**Hornerin**			+			+			+			+						
**Inositol-trisphosphate 3-kinase B**															+			
**JHDM1D protein**								+										
**Keratin 1**						+								+	+			+
**Keratin, type I cytoskeletal 10**						+												+
**Keratin, type I cytoskeletal 14**						+						+						+
**Keratin, type I cytoskeletal 16**																		+
**Keratin, type I cytoskeletal 9**						+												
**Keratin, type II cytoskeletal 2 epidermal**						+												
**Keratin, type II cytoskeletal 2 oral**																		+

**Keratin, type II cytoskeletal 6B**						+												
**Keratin, type II cytoskeletal 6C**																		+
**Keratin, type II cytoskeletal 73**						+												
**KI67 Antigen**											+							
**Kinesin-like protein KIF15**											+							
**Leukocyte-associated immunoglobulin-like receptor 1**											+							
**Maltase-glucoamylase**			+															
**Mediator of DNA damage checkpoint protein 1**										+								
**Membrane-associated guanylate kinase, WW and PDZ domain-containing protein 3**					+													
**MICOS complex subunit MIC60**											+							
**Microtubule cross-linking factor 1**						+						+						
**Mitogen-activated protein kinase kinase kinase 4**							+				+							
**MMS19 nucleotide excision repair protein homolog**				+														
**Monocarboxylate transporter 1**									+									
**Mothers against decapentaplegic homolog 6**																	+	+
**Myoglobin**								+										
**Myosin-9**					+													
**N-acetylgalactosamine-6-sulfatase**			+															
**N-acylsphingosine amidohydrolase 1**			+															
**NAD-dependent protein lipoamidase sirtuin-4, mitochondrial**		+		+										+		+		
**NADH-ubiquinone oxidoreductase chain 2**								+										
**Nanos homolog 3**											+							
**Netrin receptor UNC5D**																	+	
**Neuronal acetylcholine receptor alpha 4**		+		+														

**subunit**																		
**Neutrophil defensin 1**															+			
**Nicastrin**									+		+							
**Nuclear receptor coactivator 1**											+							
**p53-induced death domain-containing protein 1**					+													
**Partner and localizer of BRCA2**										+	+							
**peroxiredoxin-5, mitochondrial**		+						+										
**Phosphatidylinositol 4-phosphate 5-kinase type-1 alpha**						+												
**Phospholipid-transporting ATPase 8AI**			+															
**piRNA biogenesis protein EXD1**																	+	
**Probable E3 ubiquitin-protein ligase HERC1**					+													
**Protein NDRG4**					+													
**Protein phosphatase 1, catalytic subunit, alpha isoform**																	+	
**Protein S100-A8**		+	+		+			+	+		+			+	+		+	
**Protein timeless homolog**										+	+							
**PSME2 protein**											+							
**Rapamycin-insensitive companion of mTOR**					+	+												
**Receptor-type tyrosine-protein phosphatase beta**															+			+
**Resistin**															+			
**Retinol dehydrogenase 14**		+						+				+						
**Ribosomal RNA processing protein 1 homolog B**																	+	
**RNA polymerase II-associated protein 3**					+													
**Serine/threonine-protein kinase 10**									+									
**Serine/threonine-protein kinase mTOR**				+	+													
**Serine/threonine-protein kinase SMG1**				+														

**Smoothelin**						+												
**Sorbitol dehydrogenase**		+																
**Suppression of tumorigenicity 18 protein**																	+	
**Tetratricopeptide repeat protein 28**					+													
**Thymosin beta-4**						+												
**TIR domain-containing adapter molecule 1**													+		+		+	
**Topoisomerase (DNA) II binding protein 1**										+	+							
**Transient receptor potential cation channel subfamily M member 2**														+				
**Transmembrane protein 199**		+																
**Tryptophan 2,3-dioxygenase**														+				

Supplementary Table 2. Identification of proteins associated with NO synthesis, oxidative stress, neutrophil regulation, nucleic acid damage, cell survival and tissue regeneration in GCF samples obtained from patients undergoing dental bleaching with 35% H_2O_2. Samples were collected prior (baseline) and at different time-points after the bleaching process initiated.

Protein	Baseline						7 days						21 days					
	NO synthesis	Oxidative stress	Neutrophil regulation	Nucleic acid damage	Cell survival	Tissue regeneration	NO synthesis	Oxidative stress	Neutrophil regulation	Nucleic acid damage	Cell survival	Tissue regeneration	NO synthesis	Oxidative stress	Neutrophil regulation	Nucleic acid damage	Cell survival	Tissue regeneration
60S ribosomal protein L28																	+	
Abnormal spindle-like microcephaly-associated protein											+						+	
Alternative protein COMP																	+	
Alternative protein F7																		+
Alternative protein HEPACAM2					+													
Ataxin-2						+												
ATP-dependent DNA helicase				+						+						+		
Autophagy-related protein 16-1																	+	
B-cell lymphoma/leukemia 11B					+	+												
Bcl-2-binding component 3																	+	
Beta-defensin 119			+						+									
Bloom syndrome protein					+													
Calcium/calmodulin-dependent protein kinase type 1D			+															

**cDNA FLJ32673 fis, clone TESTI1000138, highly similar to Proprotein convertase subtilisin/kexin type 4 (EC 3.4.21.-)**	+																	
**cDNA FLJ35792 fis, clone TESTI2005759, highly similar to Amphoterin-induced protein 2**											+						+	
**cDNA FLJ42089 fis, clone TESOP2001166, highly similar to Suppressor of cytokine signaling 4**						+												
**cDNA FLJ50692, highly similar to RAD51-associated protein 1**				+														
**cDNA FLJ53267, highly similar to Optineurin**																	+	
**cDNA FLJ53558, highly similar to Protein disulfide-isomerase A3 (EC 5.3.4.1)**		+																
**cDNA FLJ77630, highly similar to Homo sapiens BPY2 interacting protein 1, mRNA**											+							
**cDNA FLJ78763**					+													
**cDNA, FLJ96396, highly similar to Homo sapiens ERO1-like beta (S. cerevisiae) (ERO1LB), mRNA**		+																
**Centromere protein C**					+													
**Centrosomal protein 110kDa**											+							
**Collagen alpha-3(IV) chain**											+	+						
**Cytochrome c oxidase subunit 7A2, mitochondrial**											+							
**Cytochrome P450 2A6**		+																
**Cytospin-A**																		
**Dehydrogenase/reductase SDR family member 4-like 2**		+																
**DNA repair protein-complementing XP-C cells**																+		
**DNA-binding protein Ikaros**						+												

**DNA-binding protein inhibitor ID-1**						+												
**Dual-specificity protein kinase TTK**											+							
**Dynactin subunit 1**					+													
**E3 ubiquitin-protein ligase CHFR**					+													
**E3 ubiquitin-protein ligase PDZRN3**						+												
**E3 ubiquitin-protein ligase RBBP6**										+								
**EGLN3 protein**		+						+						+				
**Elongation factor 1-alpha 2**					+						+						+	
**Enhancer of filamentation 1**																	+	
**Eosinophil cationic protein**									+									
**Epididymis secretory protein Li 55**		+						+						+				
**Fibronectin type III and SPRY domain-containing protein 1**					+													
**Flavin-containing monooxygenase**														+	+			
**Folliculin-interacting protein 2**				+						+								
**G2/mitotic-specific cyclin-B3**											+							
**Gasdermin-like isoform GSDML1**					+													
**Histone cluster 1, H1t**				+														
**Histone H4**										+								
**Histone lysine demethylase PHF8**								+										
**Homeo box A3**						+												
**Hornerin**			+			+			+			+						
**Hydroxyacyl-coenzyme A dehydrogenase, mitochondrial**														+				
**Immunoglobulin heavy variable 3-11**			+															
**Immunoglobulin heavy variable 3-23**									+									
**Immunoglobulin superfamily member 10**																		+
**Immunoglobulin superfamily member 2**															+			
**Interleukin-1 receptor type 1**			+						+									

Keratin 1														+	+			+
Keratin, type I cytoskeletal 10												+						
Keratin, type I cytoskeletal 14												+						
Keratin, type I cytoskeletal 23																		+
Keratin, type I cytoskeletal 9						+						+						+
Keratin, type II cytoskeletal 2 epidermal						+						+						
Keratin, type II cytoskeletal 3												+						
Keratin, type II cytoskeletal 6B												+						
Keratin, type II cytoskeletal 78												+						
Keratin-associated protein 5-1						+												+
Keratin-associated protein 5-11						+												
Leucine-rich repeat protein SHOC-2																		+
Leucine-rich repeat serine/threonine-protein kinase 1						+												
Meiosis-specific kinetochore protein					+													
Microtubule cross-linking factor 1					+						+						+	
Microtubule-actin cross-linking factor 1, isoforms 1/2/3/5												+						
Mitochondrial fission 1 protein					+													
MTCH1 protein											+							
Mucin-1																+	+	
Mutant xeroderma pigmentosum group C										+						+		
Myocardin					+	+												
NADH dehydrogenase [ubiquinone] flavoprotein 3, mitochondrial							+	+					+	+				
NADH-ubiquinone oxidoreductase subunit 5							+	+										
Nanos homolog 3											+							
Neutrophil defensin 1									+									
NT-3 growth factor receptor					+													

**Nuclear factor NF-kappa-B p105 subunit**			+		+													
**Nuclear protein 1**					+	+												
**Nuclear receptor coactivator 1**					+													
**Nucleolar and coiled-body phosphoprotein 1**																	+	
**O(6)-methylguanine-induced apoptosis 2**					+													
**Paired box protein Pax-1**						+												
**Partitioning defective 3 homolog**											+							
**PH domain leucine-rich repeat-containing protein phosphatase 1**																	+	
**Pleckstrin homology domain-containing family M member 1**																	+	
**Poly [ADP-ribose] polymerase 4**										+	+							
**PRAME family member 14**																	+	
**Probable E3 ubiquitin-protein ligase HERC1**											+							
**Probable ribonuclease ZC3H12D**					+													
**Protein ALEX**						+												
**Protein disulfide-isomerase A6**														+			+	
**Protein phosphatase 1, catalytic subunit, alpha isoform**					+													
**Protein rogdi homolog**						+												
**Protein S100**															+			
**Protein S100-A8**											+				+		+	
**Protein scribble homolog**					+													
**Ras-like protein family member 11B**						+												
**Retinol dehydrogenase 14**								+				+						
**Ribosomal RNA processing protein 1 homolog B**					+													
**RNA-binding protein with multiple splicing 2**																		+
**Serine/threonine-protein kinase 10**			+		+													
**Serine/threonine-protein kinase greatwall**										+	+							

**Serine/threonine-protein phosphatase 2A 56 kDa regulatory subunit gamma isoform**												+						
**Serine-protein kinase ATM**											+							
**Seven transmembrane helix receptor**						+												
**SH2 domain-containing adapter protein B**											+	+						
**Sorting nexin-18**					+													
**Spectrin beta chain, non-erythrocytic 1**					+													
**Speedy protein C**											+							
**Structure-specific endonuclease subunit SLX4**				+	+													
**Telomere-associated protein RIF1**																+	+	
**Thymosin beta-4**			+															
**Transcription activator BRG1**											+							
**TRIO and F-actin-binding protein**																	+	
**Tyrosine-protein kinase receptor**			+															
**Tyrosine--tRNA ligase, cytoplasmic**																	+	
**Ubiquitin carboxyl-terminal hydrolase 3**				+						+								
**Uncharacterized protein KIAA1109**						+						+						+
**WD repeat-containing protein 81**									+									
**Zinc finger and BTB domain-containing protein 38**				+														
